# Lentil-Based Yogurt Alternatives Fermented with Multifunctional Strains of Lactic Acid Bacteria—Techno-Functional, Microbiological, and Sensory Characteristics

**DOI:** 10.3390/foods11142013

**Published:** 2022-07-07

**Authors:** Theresa Boeck, Lilit Ispiryan, Andrea Hoehnel, Aylin W. Sahin, Aidan Coffey, Emanuele Zannini, Elke K. Arendt

**Affiliations:** 1 School of Food and Nutritional Sciences, University College Cork, T12 K8AF Cork, Ireland; theresa.boeck@umail.ucc.ie (T.B.); lispiryan@ucc.ie (L.I.); andrea.hohnel@ucc.ie (A.H.); aylin.sahin@ucc.ie (A.W.S.); e.arendt@ucc.ie (E.K.A.); 2 Department of Biological Sciences, Munster Technological University, T12 P928 Cork, Ireland; aidan.coffey@mtu.ie; 3 APC Microbiome Ireland, University College Cork, T12 K8AF Cork, Ireland

**Keywords:** fermentation, *Leuconostoc*, *Lactobacillus*, pulses, legumes, plant-based, dairy alternative

## Abstract

A milk-alternative produced from lentil protein isolate was fermented with three multifunctional strains of lactic acid bacteria, *Leuconostoc citreum* TR116, *Leuconostoc pseudomesenteroides* MP070, and *Lacticaseibacillus paracasei* FST 6.1. As a control, a commercial starter culture containing Streptococcus thermophilus was used. The metabolic performance of these strains and the techno-functional properties of the resulting yogurt alternatives (YA) were studied. Microbial growth was evaluated by cell counts, acidification, and carbohydrate metabolization. The structure of the YA was investigated by textural and rheological analyses and confocal laser scanning microscopy (CLSM). Production of antifungal compounds, the influence of fermentation on the content of FODMAPs, and typical metabolites were analyzed, and a sensory analysis was performed. The results revealed an exponential microbial growth in the lentil base substrate supported by typical acidification, which indicates a suitable environment for the selected strains. The resulting YA showed a gel-like texture typical for non-stirred yogurts, and high water holding capacity. The tested strains produced much higher levels of antifungal phenolic compounds than the commercial control and are therefore promising candidates as adjunct cultures for shelf-life extension. The *Leuconostoc* strains produced mannitol from fructose and could thus be applied in sugar-reduced YA. Preliminary sensory analysis showed high acceptance for YA produced with *Lacticaseibacillus paracasei* FST 6.1, and a yogurt-like flavor not statistically different to that produced by the control. Overall, each tested strain possessed promising functionalities with great potential for application in fermented plant-based dairy-alternatives.

## 1. Introduction

In a time where a growing world population on the one hand and the effects of climate change on the other pose a threat to food security, it is imperative to shift from excessive consumption of animal-derived foods produced by unsustainable agri-food practices to a diet higher in plant-based alternatives [1,2]. Inclusion of lentil into crop rotation systems can increase productivity and system stability [3], and milk-alternatives based on lentil protein isolate have been shown to have a carbon footprint three times smaller than that of cow’s milk [4]. The trend to replace dairy with plant-based alternatives continues to move upwards, with annual market growth rates of 11% in the EU [5]. The main objective of this research was to characterize the fermentation behavior of LAB strains that had not previously been used for the fermentation of a YA, and investigate the rheological and textural properties of the YA produced. The matrix, an emulsion containing lentil protein isolate has a carbon footprint four times smaller than that of whey protein [6], and has been previously shown to form yogurt-like gels upon acidification by fermentation with commercial starter cultures [7]. Commercial plant-based dairy alternatives are often low in protein [8,9]; using plant protein isolates instead of whole plant extracts allows for controlled protein contents that can be equal to or even higher than that of dairy products [10]. According to Codex Alimentarius Standard 243–2003 [11], yogurt is defined as milk fermented with a combination of *Lactobacillus delbrueckii* subsp. *bulgaricus* and *Streptococcus thermophilus*, products labelled with ‘alternative culture yogurt’ can also contain other *Lactobacillus* spp. Most commercially available plant-based YA are also fermented using these strains as starter cultures [12,13]. However, the effect of fermentation of YA with alternative starter cultures has been subject to intensive research [14,15,16,17,18]. The investigated strains in this study were chosen for different properties that make them promising candidates as addition to starter cultures: *Leuconostoc citreum* TR116 (LcTR116) and *Leuconostoc pseudomesenteroides* MP070 (LpMP070) are polyol producers, thus having potential for sugar-reduction while maintaining sweetness [19,20] (data for LpMP070 not published). LcTR116 is also a known producer of antifungal phenolic compounds and thus might increase shelf-life [21]. Strains of *Lacticaseibacillus paracasei* are often used as an adjunct culture for cheese ripening due to their proteolytic and aroma-producing properties [22], and preliminary sensory trials of the strain *L. paracasei* FST6.1 (Lbp6.1) showed potential as a producer of dairy-like flavors (data not published).

It could be shown that fructose as the sole carbon source induces the expression of genes responsible for mannitol production in LcTR116 [23]. Furthermore, a high-fructose-containing growing medium was found to lead to high mannitol production in *L. pseudomesenteroides* [24]. To trigger mannitol formation in LcTR116 and LpMP070, the lentil emulsion substrate for the YA was therefore supplemented with fructose. To investigate possible mannitol production in Lbp6.1 and keep fermentation conditions consistent between the strains, Lbp6.1 was likewise grown on fructose-containing lentil protein emulsion. The commercial control strain was grown in a lentil protein emulsion containing sucrose as per the manufacturer’s instructions.

## 2. Materials and Methods

### 2.1. Raw Materials and Chemicals

Lentil protein isolate (LPI) was produced from commercial red lentils (*Lens culinaris*) by aqueous extraction and isoelectric precipitation and was provided by Fraunhofer Institute for Process Engineering and Packaging, Freising, Germany. A detailed description of the production process was reported Alonso-Miravalles & Jeske et al. [6]. A functional and compositional analysis of the protein isolate was conducted by Vogelsang et al. [25] (under review), an overview of the results of this analysis is presented in Table 1. Chemicals were purchased from Sigma-Aldrich (St. Louis, MO, USA) unless otherwise stated. An overview of the LAB strains used for this study and their metabolic traits is given in Table 2. The tested bacterial strains LcTR116 and LpMP070 belong to the culture collection of the Department of Biological Sciences, Munster Technological University, Ireland; Lbp6.1 was obtained from the culture collection of the Cereal Science Laboratory of University College Cork, Ireland. All tested species have qualified presumption of safety (QPS) status and are therefore considered safe to use in food [26]. Glycerol stocks stored at −80 °C were streaked onto deMan-Rogosa- Sharpe (MRS) agar (pH 5.8) containing 0.05 g/l bromocresol green. Incubation was performed anaerobically at 30 °C for 48 h. As a control, Yoflex^®^ YF-L01 DA (Chr. Hansen, Hørsholm, Denmark), a commercial mix of two *Streptococcus thermophilus* strains marketed for the fermentation of dairy alternatives, was used.

### 2.2. LPI Emulsion Production

An LPI emulsion was produced using a modified protocol from Jeske et al. [27]. Briefly, LPI and either sucrose or fructose were added to distilled water and the LPI hydrated on a water bath at 50 °C for 1 h while stirring on a magnetic stirrer every 15 min. This was followed by shearing with an Ultra-Turrax T18 equipped with a S18N-19G dispersing element (IKA Labortechnik, Janke and Kunkel GmbH, Staufen, German) for 15 min at 6000 rpm while simultaneously stirring on a magnetic stirrer. Sunflower oil was added and sheared again for 15 min. Finally, the emulsion was homogenized in two passes using a two-stage high-pressure homogenizer (APV-2000, SPX FLOW Inc., Charlotte, NC, USA) at 800 bar (1st stage at 100 bar). The emulsion was then pasteurized at 85 °C for 2 min in a stirring water bath (Lochner mashing device, LP electronic, Berching, Germany). The final emulsions contained 3.5% protein, 1.5% oil and 5% of sucrose for the control or fructose for the tested LAB strains.

### 2.3. Lentil YA Production

A cell suspension of the tested strains was prepared by inoculating 10 mL MRS broth with a single colony and incubating at 30 °C for 24 h. From this pre-inoculum, 100 µL were sub-cultured into 10 mL fresh MRS broth and incubated for further 24 h. The concentration of colony forming units (CFU) was measured by optical density at a wavelength of 600 nm using a UV-VIS spectrophotometer (Genesys 50, Thermo Scientific, Waltham, MA, USA). The amount of inoculum necessary to reach 7 log CFU per g lentil emulsion was centrifuged at 4500 rpm for 5 min, the pellet was washed in 10 mL sterile tap water and centrifuged again. The resulting pellet was suspended in fructose-containing lentil emulsion heated to 30 °C, shaken manually for 5 min, transferred to sterile containers and fermented for 12 h at 30 °C. For the control, the necessary amount of frozen Yoflex culture to reach 7 log CFU per g lentil emulsion was added to sucrose-containing lentil emulsion heated to 42 °C, shaken manually for 5 min, transferred to sterile containers and fermented for 12 h at 42 °C. The YA were stored at 6 °C for 12–16 h prior to further analyses. Each fermentation was performed in three biological replicates.

### 2.4. Evaluation of Microbial Growth and Acidification

After inoculation and 12 h fermentation, the microbial cell count was evaluated by suspending 1 g of sample in 9 mL sterile Ringer’s solution and preparing a decimal dilution series as described by Sahin et al. [20]. Dilutions of the three tested strains were plated on MRS agar containing 0.05 g/L bromocresol green and incubated anaerobically for 48 h at 30 °C. The Yoflex control was plated on GM17 agar containing 0.5% glucose to facilitate *Streptococcus* growth and incubated anaerobically at 42 °C for 48 h. CFU/g were calculated based on the weighted arithmetic average of the counted colonies. The pH and total titratable acidity (TTA) of the samples were measured after inoculation and 12 h fermentation. TTA was measured as described by Boeck et al. [7] using titration with 0.1 M NaOH to a pH of 8.5. The concentration of organic acids in the unfermented emulsions and after 12 h fermentation was evaluated by high performance liquid chromatography (HPLC) as described by Zannini et al. [14]. Briefly, samples were diluted and filtered with a 0.2 μm syringe filter and analysed on a ) a Dionex Ultimate 3000 system (Thermo Fisher Scientific, Waltham, MA, USA) with ultraviolet light/diode array detection (UV/DAD, quantification at 210 nm; Thermo Fisher Scientific). Analytes were separated on a Hi-Plex H column (300 × 7.7 mm, Agilent, Santa Clara, CA, USA) at isocratic conditions with 5 mM sulfuric acid and 0.5 mL/min flow rate. For quantification, an external lactic and acetic acid standard in concentrations between 0.03–6 g/L was used. Each fermentation replicate was prepared for analysis in a duplicate.

### 2.5. Determination of FODMAP and Sucrose Content

FODMAP and sucrose content of the fermented and unfermented samples was measured by high-performance anion-exchange chromatography with pulsed amperometric detection (HPAEC-PAD) on a Dionex™ ICS-5000 + system (Sunnyvale, CA, USA) as described by Ispiryan et al. [28]. For chromatographical analysis, samples were diluted appropriately and filtered through 0.2 µm syringe driven filters. External calibrations with reference standards in the ranges 0.05–1 mg/L and 1–20 mg/L were applied. Xylitol, sortbitol, mannitol, glucose, fructose and sucrose were separated on the Thermo Scientific Dionex CarboPac PA1 column (2 × 250 mm) and lactose, raffinose/stachyose and verbascose on the CarboPac PA200 column (3 × 250 mm) applying isocratic and gradient elution conditions, re-spectively, as previously reported [28]. Fructans were quantified after enzymatic hy-drolysis of the diluted and filtered samples [28]. Each fermentation replicate was pre-pared for analysis in a duplicate.

### 2.6. Rheological and Tribological Measurements

The rheological properties of the YA were analyzed using a controlled stress rheometer (MCR301, Anton Paar GmbH, Graz, Austria) equipped with a concentric cylinder measuring system (C-CC27-T200/SS, Anton Paar) for the three interval thixotropy test and structure formation analysis, and a BC-12.7 ball-on-3-pins attachment with a glass ball and polydimethylsiloxane (PDMS) pins (Anton Paar) for tribology measurements. Data was evaluated using the RheoCompass software (Anton Paar, Graz, Austria).

#### 2.6.1. Three Interval Thixotropy Test

The three interval thixotropy test was performed as described by Klost & Drusch [29], with slight modifications. Analysis was performed at a sample and rheometer temperature of 6 °C, and samples were stirred 10 times with a spatula before transferring to the rheometer cup.

Oscillation was applied for 120 s at a strain of 0.1% and a frequency of 1 Hz, followed by rotational deformation at a shear rate of 200 s^−^^1^ for 200 s, and subsequent oscillation for 600 s. The oscillation parameters lie within the viscoelastic regime as established by amplitude sweeps (data not shown). The loss of structure by the differences in storage and loss modulus was calculated using the following formulas:

ΔG′ (%) = 100 − (G′_end_ × 100)/G′_start_) (1)

ΔG″ (%) = 100 − (G″_end_ × 100)/G″_start_) (2)

#### 2.6.2. Structure Formation

To measure the structure formation process of the protein gels, fermentation was performed in the rheometer while small-deformational oscillation analysis was conducted using a method described by Boeck et al. [7], with slight modifications. Briefly, the inoculated milk was transferred to the sanitized, pre-warmed rheometer cup (optimal fermentation temperature, see Table 2) and covered with a thin layer of sunflower oil to avoid evaporation. Then, during the 12 h fermentation, oscillatory analysis at a frequency of 1 Hz and a strain of 0.1% was performed, with measurements taken every 120 s.

#### 2.6.3. Tribological Analysis

**Figure 1:**
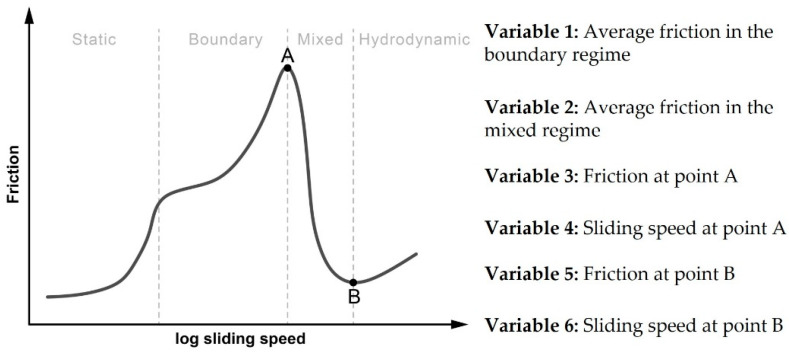
Depiction of a typical Stribeck curve, indicating points A and B for the quantification of variables, and definition of quantified variables [31,32].

The evaluation of lubrication properties of the fermented samples was performed by tribological analysis. The PMS pins were exchanged after each measurement to avoid errors due to material wear-down. The equipment was de-fatted with acetone prior to use and handled with gloves only. Samples were equilibrated to room temperature for 1 h before analysis, and rheometer temperature was set to 35 °C to mimic the temperature of the mouth cavity. A normal force of 2 N was set, representing moderate force during oral processing [30]. For analysis, 1.8 g of the fermented sample was transferred onto the sample holder and spread lightly to cover the surface of the pins. Samples were left to settle for 2 min, followed by a speed ramp-up from 10^−^^8^ to 1 m/s while recording the friction factor. All measurements were taken in triplicates for each of the three independent fermentation replicates. The differences in tribological behavior between the samples were quantified using a method described by Di Cicco et al. [31] and Fox et al. [32]. The quantifiable variables in the Stribeck curves derived from the measurements shown in Figure 1.

### 2.7. Textural Properties

The textural parameters firmness, consistency, cohesiveness and viscosity index of the YA were measured by a penetration and back-extrusion test according to a method described by Silva & O’Mahony [33]. A TA-XT2i Texture Analyzer (Stable Micro Systems, Surrey, UK) with a 25 kg load cell and an extrusion disc (Ø = 35 mm) was used at a probe speed of 1 mm/sec, a penetration depth of 20 mm and a trigger force of 0.05 N. Samples for texture measurements were fermented in individual sterile 200 mL cups, and analysis was performed 12–16 h after fermentation at a sample temperature of 6 °C, without stirring of the samples before measurements.

### 2.8. Water Holding Capacity

A centrifugation method as described by Grasso et al. [8] was used to determine water holding capacity of the YA. For analysis, 30 g of the samples fermented in sterile centrifugation tubes were centrifuged at 640× *g* for 20 min at 5 °C, and the water holding capacity (WHC) calculated using the formula

WHC [%] = (1 − mass of supernatant/mass of sample) × 100 (3)

### 2.9. Whiteness Index

The color values of the fermented and unfermented samples were measured with a Chroma Meter (Minolta CR-400, Osaka, Japan) using the D65 illuminant. The CIE L*a*b* coordinates were obtained, with L* representing black (0) to white (100), a* representing green to red (negative values are green, positive are red), and b* representing blue to yellow (negative values are blue, positive are yellow). These values were used to calculate the whiteness index (WI), using the equation:

WI = 100 − √((100 − L*)^2^ + a*^2^ + b*^2^) (4)

### 2.10. Ultrastructure of YA

The microstructure of the samples was analysed with a FV1000-IX81 confocal laser scanning microscope (Olympus, Tokio, Japan) with a He-Ne laser (excitation wavelength 633 nm, emission detection between 565–615 nm). For a visualization of the undisturbed gel structure a modified protocol from Hickisch et al. [34] was used. After inoculation, samples were mixed with an aqueous 0.001% (*w/v*) solution of Nile Blue A Perchlorate to a concentration of 5% (*v/v*) of dye solution per sample and fermented in tissue culture chambers (Sarstedt, Nuembrecht, Germany). The unfermented samples were mixed with dye to the same concentration and analysed directly. Microscopy was performed on the tissue culture chambers with 60× magnification.

### 2.11. Quantification of Antifungal Compounds

The samples were screened for the presence of compounds with antifungal properties using a method by Hoehnel et al. [21]. The fifteen compounds (benzoic acid, 4-hydroxybenzoic acid, caffeic acid, hydrocaffeic acid, catechol, ferulic acid, hydroferulic acid, hydrocinnamic acid, methylcinnamic acid, 3-phenyllactic acid, 4-hydroxyphenyllactic acid, phloretic acid, p-coumaric acid, salicylic acid, vanillic acid) were chosen for their reported antifungal activity and presence in lactic acid bacteria fermentates [35,36]. Extractions were carried out as described by Brosnan et al. [37] with modifications. Briefly, the frozen samples were freeze dried, ground to a powder with a mortar and pestle, and defatted three times with 10 mL petroleum ether per 1 g freeze-dried sample. For calculations, the moisture content of the fresh and de-fatted freeze-dried samples was determined with the air-oven method AACC method 44–15.02 [38]. Then, 2 g of the de-fatted freeze-dried sample was mixed with 10 mL of ultrapure water and 10 mL ethyl acetate containing 0.1% (*v/v*) formic acid using a vortex mixer. Next, 1 g sodium chloride and 4 g magnesium sulfate were added and the samples mixed on a vortex mixer for 1 min. After centrifugation for 10 min at 4800× *g*, the organic phase (top layer) was transferred to solid-phase extraction tubes (Bond Elut QuEChERS Dispersive kit; Agilent Technologies Inc., Santa Clara, CA, USA), homogenized on a vortex mixer and centrifuged for 10 min at 2300× *g*. Then, 5 mL of the supernatant were transferred to centrifugation tubes containing 100 µL dimethylsulfoxide (DMSO) and the solvents evaporated using a vacuum centrifuge (Scanvac Scanspeed, Labogene, Lillerød, Denmark) for 3 h at 500 rpm and 45 °C. After reconstituting the concentrated extract with 400 µL ultrapure water/acetonitrile 90/10 (*v/v*), the extracts were filtered using 0.2 µm syringe filters. For chromatographic analysis, a Dionex UltiMate 3000 RSLC system (Thermo Fisher Scientific, Waltham, MA, USA) equipped with a Gemini C18 column (2 × 150 mm; Phenomenex Inc., Torrance, CA, USA), matching guard cartridge and a UV/DAD detector (Thermo Fisher Scientific, Waltham, MA, USA) was used. The analysis parameters were described by Hoehnel et al. [21]. For quantification, an external calibration with reference standards of the compounds (5–50 ppm, 5–245 ppm for phenyllactic acid) was applied.

### 2.12. GC-MS Metabolomics

Freeze-dried samples were reconstituted in water at a volume of four times the mass of the sample, centrifuged at 16,000× *g* for 30 min and the supernatant transferred to a new tube. This centrifugation step was repeated twice and the extract then filtered using centrifuge filters (Merck Ultrafree-CL GV 0.22 µm). Analysis of the derivatizated compounds with methyl chloroformate was carried out by MS-Omics, Denmark, using gas chromatography coupled with a quadropole mass spectrometry detector (GC-MS, Agilent) following a slightly modified version of the method described in Smart et al. [39], and gas chromatography coupled with a quadropole mass spectrometry detector (GC-MS, Agilent). Samples were analyzed for several typical metabolite compounds from the tricarboxylic acid cycle, i.e., pyruvic acid, succinic acid, fumaric acid, malic acid, α-ketoglutaric acid, cis-aconitic acid, citric acid, isocitric acid, 4-aminobutyric acid (GABA), and malonic acid. Data were evaluated using Chemstation (Agilent) and Matlab R2018b (Mathworks Inc., Natick, MA, USA).

### 2.13. Sensory Analysis

Sensory analysis of the fermented samples was performed by a panel of experienced sensory testers (n = 13, age range 23–29) recruited from the Department of Food Science at the University College Cork. Panelists were asked to rate the intensity on a scale from 0 to 10 of the following descriptors: Odor Intensity, yogurt-like odor, flavor intensity, sourness, bitterness, beany flavor, yogurt-like flavor, sour-cream like flavor, firm mouthfeel, smooth mouthfeel, and aftertaste. Panelists were also asked to rate the overall acceptability of the samples on a scale from 0 to 10. The questionnaire including the definitions of the descriptors used for the sensory testing can be found in the Supplementary Materials Figure S1. Sensory analysis was conducted in a sensory analysis room and were performed in duplicate on two separate days.

### 2.14. Statistical Analysis

All analyses were performed in three independent fermentation trials. One-way analysis of variance (ANOVA) and post-hoc Tukey test (*p* ≤ 0.05) were performed to determine significant differences between groups. When according to Levene’s test, equal variances were not assumed, Welch’s *t*-test and Games-Howell post-hoc test (*p* ≤ 0.05) were used instead. All statistical analyses were performed using IBM SPSS Statistics, version 28.

## 3. Results and Discussion

### 3.1. Microbial Growth, Acidification and Color of the YA

**Figure 2:**
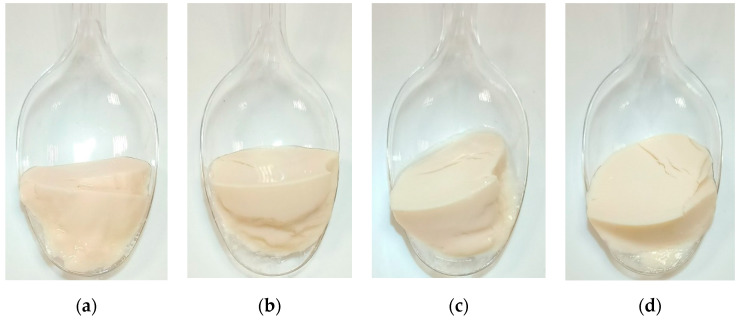
Appearance of the fermented YA, photographed after 12 h storage at 6 °C, removed from the fermentation container. (**a**) Yoflex control, (**b**) LcTR116, (**c**) LpMP070, (**d**) Lbp6.1.

The appearance of the fermented YA is illustrated in Figure 2. LcTR116 and LpMP070 showed the highest growth in the lentil emulsion medium, resulting in a final cell count of 1.12 × 10^9^ and 3.69 × 10^9^ CFU/g, respectively (Table 3), while the growth of Lbp6.1 was lower (4.50 × 10^8^ CFU/g). Interestingly, the Yoflex control showed the weakest growth, with an increased cell count by one log and a final concentration of 2.71 × 10^8^ CFU/g after 12 h fermentation time. Other studies on plant-based YA reported similar LAB colony counts at the end of fermentation. Jiménez-Martínez et al. [40] obtained 3.2 × 10^8^ CFU/mL in an 8 h fermentation of a lupin-based yogurt like product with *S. thermophilus* and *Lb. delbrueckii* ssp *bulgaricus;* Zannini et al. [14] produced a lentil-based YA by fermentation with *Weissella cibaria* MG1 and reported 1.31 × 10^9^ CFU/mL after 24 h fermentation.

The pH of the unfermented lentil emulsions was 6.92 ± 0.10 at a TTA of 0.08 ± 0.01 mL 0.1 M NaOH per g of sample (Table 3). After 12 h of fermentation, the pH of the Yoflex control dropped to 4.12 ± 0.02. Fermentation with Lbp6.1 resulted in a slightly higher pH than the Yoflex control (4.41 ± 0.06), while the pH values of the yogurts fermented with LcTR116 and LpMP070 did not significantly differ from the control. The TTA of the LpMP070 and LcTR116 samples was significantly higher than that of the Yoflex control (0.86 ± 0.07 and 0.64 ± 0.01 mL, respectively), while the TTA of the YA fermented with Lbp6.1 was significantly lower (0.39 ± 0.02 mL). These discrepancies between TTA and pH can be explained by the differences in the acid dissociation constant (pKa) and thus different buffering capacities of the acids produced by the different strains [41,42]. Both Yoflex and Lbp6.1 are homofermentative, so lactic acid is the main acid produced, while LcTR116 and LpMP070 are heterofermentative and produce both lactic and acetic acid. As measured by HPLC, LcTR116 produced 3.36 ± 0.03 g lactic acid per kg of YA, and LpMP070 produced 3.63 ± 0.03 g lactic acid and 1.90 ± 0.02 g acetic acid per kg. Lbp6.1 produced 3.68 ± 0.03 g/kg lactic acid, and the Yoflex control 4.58 ± 0.05 g/kg lactic acid. Lactic acid has a pKa of 3.86, while acetic acid is a weaker acid at a pKa of 4.75 [43]. The pH is therefore more strongly influenced by lactic acid than acetic acid content [24].

Dairy yogurt pH typically lies around 4.5, but commercial yogurts with a pH as low as 3.7 have been reported [44]. A study on 6 commercial plant-based YA reported a pH between 3.99 and 4.38, and a TTA between 0.12 and 0.78 mL NaOH/g [8]. The pH values of the YAs fermented with the tested strains compare well to those typically found in dairy yogurts and plant-based YA. The amount of lactic acid in the tested samples was found to be similar to the commercial plant-based YAs, but lower than that found in dairy yogurt (11.1 g of lactic acid/kg) [8]. The similar pH of plant-based YA and dairy yogurt while containing a lower amount of acid can be explained by the higher buffering capacitiy of the dairy proteins and salts compared to those present in the plant-based samples [45]. While the TTA of the YA fermented with LpMP70 was slightly higher than that of commercial plant-based YA, the other strains produced YA with typical TTA values.

The lentil protein isolate used in this study is pale pink due to anthocyanins and carotenoids contained in the hulls of the red lentils the protein is isolated from [46]. The lentil protein emulsions therefore also show a pink color with high a* values (Table 3). The whiteness index of the samples was increased significantly by fermentation and was increased from around 73 to 76–77. This might be due to a susceptibility to degradation of the natural lentil carotenoids in an acid environment [47] and pH-dependent color change of the anthocyanins [48]. The average WI of dairy yogurts determined to be 82.4 ± 8.1 [49], while commercial plant-based yogurts were reported to have an average WI value of 62.0 ± 1.7 [8]. The lentil-based YAs therefore show a much higher WI than other plant-based YA currently on the market.

### 3.2. Uniaxial Compression Testing

**Figure 3:**
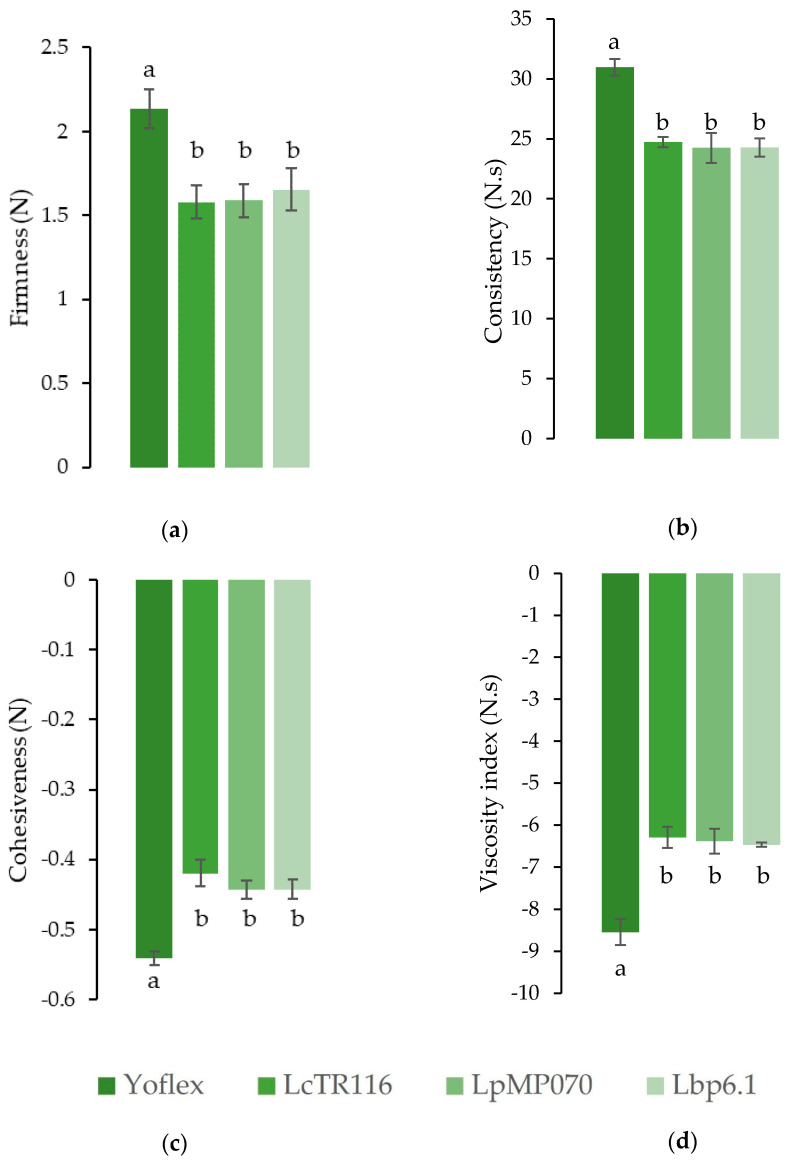
Averages and standard deviations of (**a**) firmness, (**b**) consistency, (**c**) cohesiveness, and (**d**) viscosity index of the fermented samples as measured by uniaxial compression testing. Values with the same superscript letters are not significantly different (*p* > 0.05).

Large deformation tests simulate the breakdown of food in the mouth [50]. Firmness and consistency are measured while the probe is penetrates the sample, whereas cohesiveness and viscosity index are recorded when the probe moves back to its original position. Firmness is the maximum point of the force-time curve and expresses the force necessary to break a gel structure. The positive area under the force-time curve gives the thickness of the consistency of the YA. Cohesiveness is the maximum negative force in the force-time curve to pull up the probe, and the viscosity index is the negative area of the force-time curve, representing the remaining of the sample on the probe which indicates restricted flowing behavior [50,51]. Figure 3 shows that no statistical difference in either firmness, consistency, cohesiveness or viscosity index between the experimental strains could be found. However, the three tested strains tested slightly lower than the Yoflex control in all factors, indicating that the commercial strain yields a firmer, thicker and more cohesive product. 

### 3.3. Rheological and Tribological Behavior

**Figure 4:**
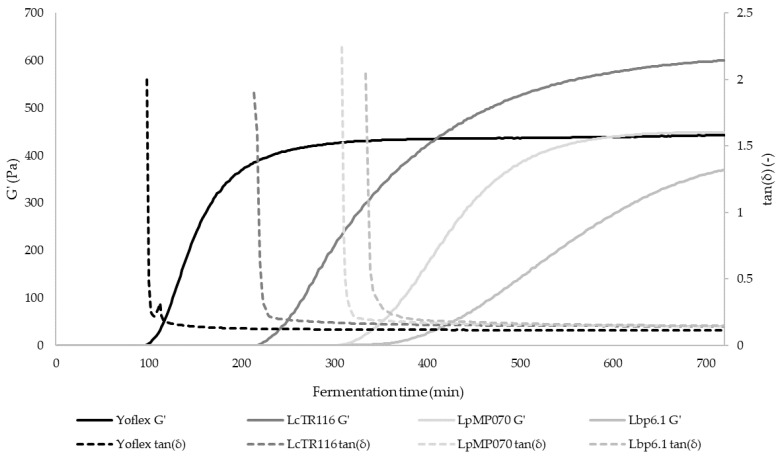
Storage modulus G′ (left *y*-axis) and damping factor tan(δ) (right *y*-axis) measured over 12 h fermentation time to monitor gel formation. Average values are displayed, error bars have been omitted for clarity.

Oscillatory analysis during fermentation in the rheometer gave insight into the different gelation kinetics of the different strains (Figure 4). Gel formation is indicated by a rise of the storage modulus G′, which represents the solid or elastic proportion of a viscoelastic material, while at the same time, the loss modulus G″, which represents the liquid or viscous proportion, decreases. The damping factor tan(δ) is the proportion of G″ to G′. When G′ is larger than G″ and thus tan(δ) < 1, the gelling point is reached, and solid behavior becomes dominant in the material [52]. Gel formation in the Yoflex control was the fastest, starting after 100.7 ± 8.3 min of fermentation time (Table 4). One of the factors influencing acidification rate is fermentation temperature [53], which was higher for Yoflex and therefore explains the faster acidification. In LcTR116, gel formation was reached after 212.8 ± 1.0 min, followed by LpMP070 after 306.1 ± 5.4 min. As both pH values and microbial cell counts at the end of fermentation are similar with both strains, the results of this test indicate a longerlag phase for LpMP070 than for LcTR116. Gelation in Lbp6.1 was the slowest at 328.3 ± 16.5 min, which can be explained by its lower microbial growth compared to the other samples.

In the three interval thixotropy test, the samples are first oscillated, then sheared and again oscillated. During the shearing, the structure of the samples is disrupted, and then partially restructures in the following oscillation step. This gives indication of the structural stability and robustness against shear stress of a gel, which are important properties during production and transportation [54]. Throughout both oscillation steps, the damping factor tan(δ) of all samples stayed < 1, so the gel state of the samples was upheld.

In the Yoflex control, 43.5% of the G’ and 53.2% of the G” was lost, which is significantly more than the tested strains (see Table 4). In LcTR116, LpMP070 and Lbp6.1, G’ values were lowered by 29.8%, 26.37% and 36.55%, respectively, G’’ decreased by 38.21%, 40.72% and 45.09% respectively. Therefore, all tested strains resulted in YA with structures less susceptible to damage by shearing than the YA produced by the commercial culture control.

Tribological analysis of the YA samples resulted in Stribeck curves similar to those reported in previous studies on yogurt (Supplementary Figure S2). While Di Cicco et al. [31] were able to discern between yogurt samples by differences in variable 2, in this study, no statistical differences could be found between samples for variables 1, 2 and 3 (average frictions of boundary and mixed regimes, and friction at point A, for values see Supplementary Table S1, for definition of variables, see Figure 1). As can be seen in Table 4, small differences between samples could be measured in variable 4 (the speed at which point A is reached), variable 5 (friction at point B) and variable 6 (the speed at which point B is reached). However, during sensory testing (Section 3.9), no significant differences in respect to firmness or smoothness of mouthfeel were reported.

### 3.4. Water Holding Capacity

**Figure 5:**
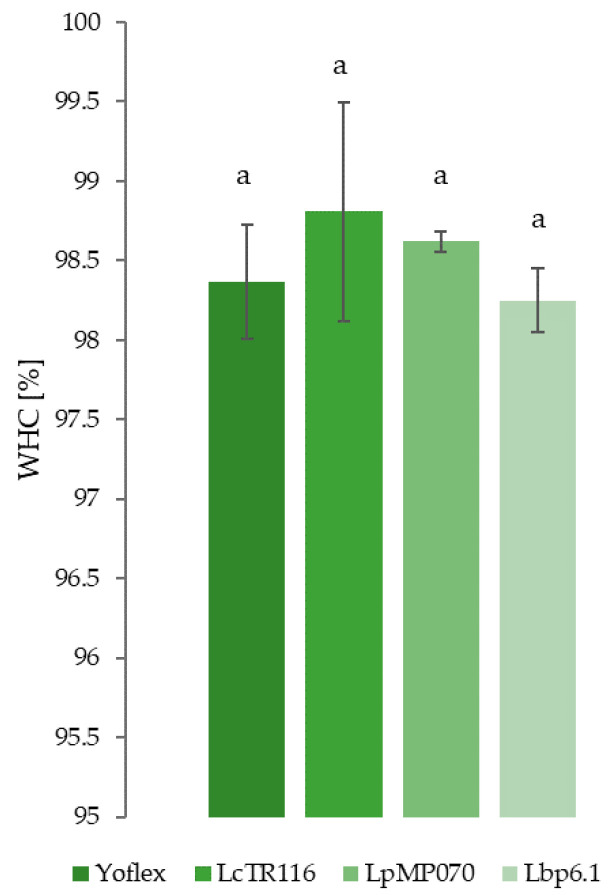
Water holding capacity (WHC) as measured by centrifugation method. Error bars represent standard deviations. Values with the same superscript letter are not significantly different (*p* > 0.05).

High water holding capacity is a crucial quality attribute of yogurt, as syneresis, i.e., a layer of whey on top of the product due to gel contraction, is undesirable by consumers [55]. Low water holding capacity also means the yogurt is susceptible to whey separation during processing and transport [56]. No statistical difference could be found between water holding capacities between the Yoflex control and the tested strains, and water holding capacity ranged between 98.30% in LpMP070 and 98.62% in Lbp6.1 (Figure 5). A study comparing several commercial plant-based YAs found water holding capacities between 99.3% and 82.8% [8]. This demonstrates that all tested strains can produce a lentil-based YA with water holding properties comparable to products already commercially available.

### 3.5. Microstructure of the Protein Gels

**Figure 6:**
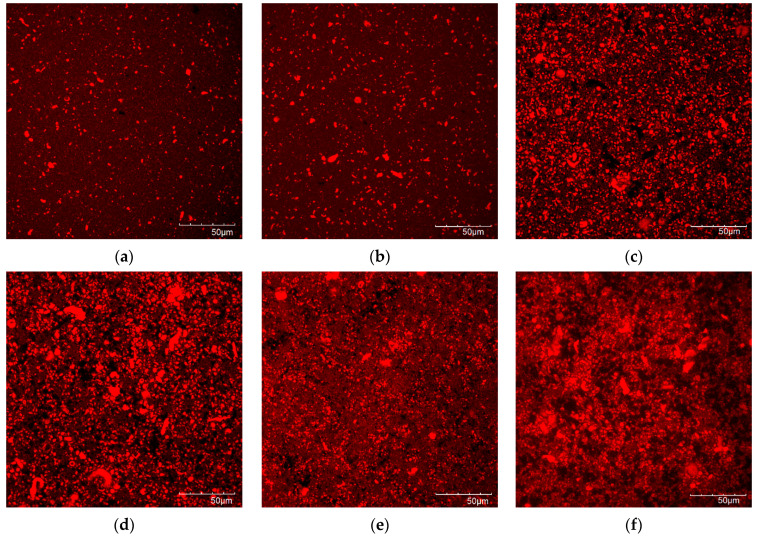
CLSM images of the unfermented and fermented samples. Nile Blue was used to stain the protein, shown in red. (**a**) unfermented sample with fructose, (**b**) unfermented sample with sucrose, (**c**) Yoflex control, (**d**) LcTR116, (**e**) LpMP070, (**f**) Lbp6.1.

The protein microstructure is shown in Figure 6. In the unfermented emulsions (a and b), a loose dispersion of protein particles can be observed. In contrast, in the fermented Yas (c–f), the protein was aggregated and formed the gel network. The protein network of the Yoflex control (c) is characterized by large pores. Fermentation using LcTR116 resulted in a protein network with larger agglomerates and slightly bigger pores compared to the control. The samples fermented with LpMP070 appear to have a protein network made of thinner strands with very small pores. The structure of the YA fermented with Lbp6.1 shows irregularly sized pores and also a relatively coarse structure. Gel coarseness of legume protein gels of the same concentration has been shown to be determined mainly by pH and ionic strength [57], and a coarser gel structure with larger pores has been shown to indicate lower gel strength [58]. However, the differences observed in CLSM in this study appear minor and do not seem to correlate with the quantified pH values or textural and rheological properties.

### 3.6. Content of FODMAPs and Sucrose

FODMAPs are a group of short-chain carbohydrates that are either insufficiently or not absorbed in the small intestine during digestion, therefore reaching the large intestine where they are osmotically active and can act as a carbon source for the gut microbiota. Common FODMAPs include fructose (which is poorly absorbed in the small intestine when in excess of glucose [59]), lactose, polyols, fructans, and α-galactooligosaccharides (GOS). While for healthy individuals, FODMAPs can be a valuable source of prebiotics [60], a diet low in FODMAPs has been shown to alleviate symptoms of patients with irritable bowel syndrome (IBS) [61]. The GOS raffinose, stachyose, and verbascose are the main FODMAP in lentil [62], however, during IEP of the protein isolates from lentil flour, the GOS content is reduced significantly [63]. The total GOS content (raffinose, stachyose and verbascose) of the unfermented samples was 0.14 ± 0.00 g per 100 g (Table 5). While fermentation with LpMP070, Lbp6.1, and Yoflex did not affect GOS levels, fermentation with LcTR116 reduced the raffinose/stachyose content by 56% from 0.10 g/100 g in the unfermented emulsion to 0.05 g/100 g after fermentation. Reduction in GOS of legume products by fermentation with LcTR116 have been observed before, but were attributed to legume enzymes rather than α-galactosidase activity of LcTR116, as *L. citreum* has been reported to be α-galactosidase negative [21,64]. Further research into the GOS metabolism of LcTR116 is needed to investigate the potential α-galactosidase enzyme activity and metabolic pathways of this strain, possibly using genetic and proteomic methods as found in Ortiz et al. [65].

The cut-off for GOS per serving in a low-FODMAP diet has been set at 0.2 g per serving [66]. In a typical serving of 150 g [67], the GOS content is exceeded in all samples (0.21 g GOS per serving), except for the sample fermented with LcTR116 (0.15 g GOS per serving).

Polyols are not naturally present in pulses and, therefore, not present in the unfermented samples. However, the heterofermentative strains LcTR116 and LpMP070 express mannitol dehydrogenase and, thus, can produce mannitol from fructose [68]. With 0.98 g mannitol per serving in the LcTR116 sample and 1.21 g/serving in the LpMP070 sample, the low FODMAP cut-off of 0.20 g mannitol per serving is exceeded approximately five- and six-fold, respectively [66]. For healthy individuals, however, conversion of fructose to mannitol is beneficial, as it is low-calorie and does not induce a glycemic response while retaining 50–70% of the sweetness of sucrose [69]. Using both glucose and fructose as a substrate for the fermentation could further increase mannitol production [70]. While ingestion of more than 20 g of mannitol per day can lead to a laxative response due to osmotic imbalances in the colon, no laxative effect is expected in healthy individuals at the levels present in the YA [71]. Fructose in excess of glucose can also cause abdominal discomfort for individuals with IBS or fructose malabsorption [72]. The low-FODMAP cut-off value for fructose has been set at 0.15 g/serving [66]. The samples fermented with LcTR116, LpMP070 and Lbp6.1, to which fructose was added as a carbon source for fermentation, exceeded this limit, while the samples fermented with Yoflex, for which sucrose was used, did not. Measurements of the fermented samples show that fermentation with LcTR116 only metabolised 24.9% of the fructose provided, LpMP070 22.9% and Lbp6.1 5.5%. In contrast, in the Yoflex control, only 0.353 g of the sucrose was metabolized, which is equivalent to 7.7% of the initial addition level. Therefore, sugar content could be reduced in future trials for the development of lower-carbohydrate products. No other FODMAPs (i.e., other polyols, lactose, fructans) were detected in any of the samples. Overall, none of the YAs met the low FODMAP criteria according to Varney et al. (cutoff levels for fructose, polyols and/or oligosaccharides exceeded) to be recommended for consumption by IBS patients with pronounced sensitivities towards these compounds [66].

### 3.7. Content of Antifungal Phenolic Acids

Of the 15 tested phenolic compounds with reported antifungal activity, six were found to be present in concentrations above the limit of quantification: phenyllactic acid, coumaric acid, salicylic acid, benzoic acid, 4-hydroxybenzoic acid, and vanillic acid (Table 6). Phenolic acids in foods possess a wide range of functionalities, including shelf life extension [73,74], antioxidative [75], antidiabetic [76] and anti-inflammatory [77] activities, and show high bioavailability [78].

Vanillic acid was present in the unfermented emulsions, but levels decreased significantly by fermentation with the tested strains. However, no decrease in vanillic acid was observed in the Yoflex control. This suggests that the tested LA B strains metabolized the vanillic acid present in the raw material, which has been observed previously in *L. pseudomesenteroides* and other LAB strains [79], and may be related to bacterial decarboxylation metabolism [80]. 4-Hydroxybenzoic acid, salicylic acid and coumaric acid were present in the unfermented samples, and levels increased slightly during fermentation. The most dominant antifungal compounds detected in the samples was phenyllactic acid. Phenyllactic acid is produced by many lactic acid bacteria species such as *Lactobacillus*, *Leuconostoc*, *Weissella*, and *Enterococcus* spp. [81], and found in fermented foods, such as pickles [82] or sourdough [83,84]. As it is metabolized from the amino acid phenylalanine [85], of which lentil protein has a high content of [86,87], lentil protein emulsion is an ideal substrate for phenyllactic acid production. Phenyllactic acid has been shown to possess strong antimicrobial properties against fungi such as *Penicillium* and *Aspergillus* spp. [73] and a wide range of bacteria, such as *Listeria monocytogenes, Staphylococcus aureus, Bacillus cereus,* and *Escherichia coli* [88]. Phenyllactic acid also was found to be a precursor in the formation of typical aromatic carboxylic acids in cheese production [85]. While not detected in the unfermented samples, it was present in all fermented YAs. The Yoflex control contained 1.55 mg/kg of phenyllactic acid, the lowest amount amongst the fermented samples. The YAs fermented with LcTR116 and Lbp6.1 contained 5.52 and 4.40 mg/kg of phenyllactic acid respectively. The sample fermented with LpMP070 contained by far the highest levels of phenyllactic acid, with a content of 8.28 mg/kg. The variations in phenyllactic acid content between samples might be due to differing expression levels of the aminotransferase and phenylpyruvate dehydrogenase between the strains that catalyze the conversion of phenylalanine to phenyllactic acid [89]. However, it should be taken into account that LcTR116 and LpMP070 had a much higher rate of microbial growth than Lbp6.1 and Yoflex, and therefore more phenyllactic acid was more likely to be produced.

### 3.8. Analysis of Metabolites

**Figure 7:**
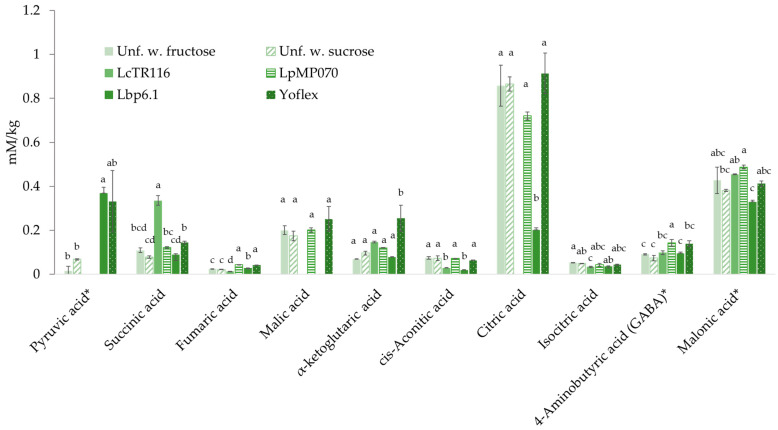
Several metabolites of the tricarboxylic acid cycle in the unfermented and fermented samples as measured by GC-MS. Error bars indicate the standard deviation. Values for each metabolite with the same superscript letters are not significantly different (*p* > 0.05). * Relative quantification, values are normalized peak areas.

While the metabolism of LcTR116 has been reconstructed based on genomic analysis [23], no metabolic pathway reconstruction has been performed on LpMP070 and Lbp6.1. and no information on metabolomic traits is publicly available on the *S. thermophilus* strains in the commercial control. With the quantification of several central metabolites of the tricarboxylic acid cycle (Figure 7), this study provides a better understanding of the metabolism of LcTR116 in a lentil protein based substrate, and provides first insights into the metabolic pathways of LpMP070 and Lbp6.1

In homolactic metabolism (Lbp6.1 and Yoflex), hexoses are metabolized via the Emden-Meyerhoff-Parnas (EMP) pathway to pyruvate, which is the main electron acceptor in LAB metabolism, which is then converted to lactic acid by lactate dehydrogenase. In heterolactic fermentation, hexoses are metabolized via the phosphoketolase pathway, producing not only lactate from pyruvate, but also CO_2,_ and acetate or ethanol as end products [90]. Pyruvate, was not detected in the samples fermented with the heterofermentative LcTR116 and, and only low levels were present in the unfermented samples, while on the other hand, higher levels were detected in the samples fermented with the Lbp6.1 and the *S. thermophilus* of the Yoflex control. This might indicate an inhibition of lactate dehydrogenase. LcTR116 also has been found to be able to metabolize pyruvate in alternative pathways, as it can express a pyruvate dehydrogenase complex. This facilitates the conversion of pyruvate to acetate via acetyl-CoA, yielding an extra ATP [23]. Whether LpMP070 also contains genes for pyruvate dehydrogenase is unknown, but it has been shown to be present in the closely related *Lc. mesenteroides* [91]

Citric acid is present in the unfermented samples, but not detected in the samples fermented with LcTR116. This strain possesses a citrate lyase complex [23], which is common in *Leuconostoc* spp. and enables the use of citrate as a carbon source [92]. The citrate lyase facilitates cleavage of citrate into oxaloacetate and acetate. LcTR116 also contains a oxaloacetate decarboxylating malic enzyme which produces pyruvate. The pyruvate can either be converted to acetate, or decarboxylated to fumarate, which is then dehydrogenated to succinate by succinate dehydrogenase [93]. Succinate is present in the LcTR116 sample in levels significantly above those of the unfermented emulsions, but is not increased in any of the other fermented YA. Citrate metabolism is also responsible for the production of several metabolites typical for the aroma of certain fermented dairy products, such as diacetyl or acetoin [92]. The YA fermented with Lbp6.1 also had significantly lower citrate levels which might indicate citrate metabolism activity, which has been previously shown in *L. casei* and *L. paracasei* [94,95]. Fumaric acid is only slightly elevated and the levels of succinic acid are not higher than those in the unfermented samples, which might indicate an absence of succinate dehydrogenase. Fumaric acid levels are also slightly elevated in the samples fermented with LpMP070 and Yoflex. Given that the citrate concentration in the samples fermented with either strain is not significantly reduced, citrate metabolism seems unlikely. It has been shown that *S. thermophilus* is able to produce fumaric acid from argininosuccinate during arginine biosynthesis [96].

Only the YA fermented with the control strain Yoflex contains α-ketoglutarate in levels above those of the unfermented samples. This compound is produced by the deamination of glutamate by glutamate dehydrogenase and is essential in amino acid catabolism. Absent glutamate dehydrogenase activity is common in many *Leuconostoc* and *Lactobacillus* spp. [93], and high activity has been reported for *S. thermophilus* [93,94]. α-ketoglutarate is the main acceptor of the amino group in amino acid transamination reactions in LAB metabolism [97]. The resulting α-ketoacids are converted into aroma compounds, e.g., aldehydes, alcohols, or carboxylic acids, which are crucial in cheese ripening [98], but can also play a role in yogurt flavor formation by acetaldehyde production [99]. α-ketoglutarate is converted to glutamate in the in the transamination step of phenyllactic acid metabolism [89]. The low α-ketoglutarate levels could therefore be attributed to the high levels of phenyllactic acid that were observed in LcTR116, LpMP070, and Lp6.1.Gamma-aminobutyric acid (GABA) is an inhibitory neurotransmitter produced by glutamate decarboxylase (GAD) from glutamate [100]. Supplementation has been shown to have various positive health impacts, such as anxiolytic [101], anti-diabetic [102] and anti-hypertensive activity [103]. GABA is naturally present at subclinical levels in many foods, and fermentation with GAD-positive strains of LAB, has been shown to increase contents significantly [104,105]. GABA levels are significantly elevated in the YA fermented with LpMP070 and Yoflex, indicating GAD activity.

### 3.9. Sensory Analysis

The overall odor intensity as well as the most yogurt-like odor was ascribed to Lbp6.1 and the Yoflex control (Table 7).

Sourness was rated highest for the sample fermented with the heterofermentative LpMP070, which was also the sample with the highest TTA. The second heterofermentative stain, LcTR116, scored the second-highest sourness levels, while the homofermentative Lbp6.1 attained very low sourness ratings in comparison. No significant differences could be found in bitterness and beany flavor between samples. The two heterofermentative strains received deficient ratings in the factor yogurt-like flavor, while Lbp6.1 and the Yoflex control scored significantly higher in this category, with no statistically significant difference between Lbp6.1 and the control. The evaluation of mouthfeel (firmness and smoothness) indicated no significant difference between either of the samples. The Yoflex control obtained the lowest aftertaste intensity, while LcTR116 and LpMP070 scored the highest. Overall acceptability was highest for the Yoflex control, followed by Lbp6.1, while the acceptability of LcTR116 and LpMP070 was significantly lower. For a more in-depth sensory characterisation from a consumer-based perspective, other sensory methods such as CATA (check-all-that-apply) questions, polarised sensory positioning, or projective mapping could prove useful [106].

## 4. Conclusions

The use of lentil protein isolate as a base material allows for producing a YA with a protein content equal to that found in dairy yogurt. Life cycle analyis of the lentil protein analysis has found the carbon footprint to be up to four times lower than that of whey protein, caseinate, and soy protein isolate [6,107], so the carbon footprint for the YA is expected to be very low, making the YA a good source of sustainable protein. However, very little is known about the fermentation characteristics of LAB in a lentil-based substrate. The present study reveals the performance of three multifunctional LAB strains when used as starter cultures for a lentil-based YA compared to a commercial LAB starter culture optimized for the fermentation of plant-based YA. It could be shown that all three tested strains grew well on the lentil emulsion substrate and were successful in producing a lentil-based YA with high whiteness index, yogurt-like textural characteristics (gel-network formation) and water holding capacities comparable to that of the control. LcTR116, LpMP070, and to a lower degree Lbp6.1 showed potential to be used as adjunct co-cultures with traditional starter cultures due to their ability to produce antifungal compounds for increased shelf life. LcTR116 and LpMP070 convert fructose to mannitol and could thus be used for sugar-reduced YA applications. Both the yogurt-like flavor and overall acceptability of Lbp6.1 were equal to the control and scored highly in the sensory tests. Overall, each of the strains was shown to possess beneficial traits for the fermentation of lentil-based dairy alternatives.

## Figures and Tables

**Table 1:** Compositional analysis and solubility of the red lentil protein isolate (from Vogelsang et al. [25]), and methods used for analysis.

**Moisture (%)**	5.9 ± 0.1	AACC Method 44-17.01
**Protein content (% DM)**	74.94 ± 0.71	Kjeldahl (Factor 6.25)
**Fat (% DM)**	5.24 ± 0.16	Soxhlet
**Ash (% DM)**	6.55 ± 0.04	AACC Method 08-01.01
**Solubility (%) ^1^**	63.26 ± 1.46	Kjeldahl

Mean values ± standard deviations. ^1^ Protein solubility at pH 7, expressed as percentage of total protein.

**Table 2:** Strains of lactic acid bacteria used in this study with relevant information.

Species	*Leuconostoc* *citreum*	*Leuconostoc* *pseudomesenteroides*	*Lacticasei-* *bacillus* *paracasei*	Control: *Streptococcus* *thermophilus*
**Strain**	TR116	MP070	FST6.1	YOFLEX^®^ YF-L01 DA ^1^
**Metabolism**	Hetero- fermentative	Hetero- fermentative	Homo- fermentative	Homo- fermentative
**Carbon source**	Fructose	Fructose	Fructose	Sucrose
**Incubation**	30 °C	30 °C	30 °C	42 °C
**Source**	Yellow pea sourdough	Kombucha	Water kefir	-
**Special Traits**	Antifungal compounds & mannitol producer	Antifungal compounds & mannitol producer	Dairy flavor producer	Marketed for fermentation of dairy alternatives

^1^ Brand name, strains not specified by manufacturer.

**Table 3:** Fermentation characteristics and color of yogurt alternatives produced with different strains.

	LcTR116	LpMP070	Lbp6.1	Yoflex	Unfermented (Fructose)	Unfermented (Sucrose)
**Fermentation** **parameters**						
T0 (CFU × 10^7^/g)	1.37 ± 0.40 ^ab^	1.12 ± 0.09 ^a^	1.22 ± 0.24 ^ab^	1.66 ± 0.27 ^b^	n.d. ^1^	n.d. ^1^
T12 (CFU × 10^7^/g)	111.85 ± 13.24 ^b^	368.89 ± 140.63 ^a^	45.03 ± 11.04 ^c^	27.07 ± 9.02 ^d^	n.d. ^1^	n.d. ^1^
pH	4.31 ± 0.01 ^bc^	4.10 ± 0.01 ^a^	4.41 ± 0.06 ^b^	4.12 ± 0.02 ^cd^	6.89 ± 0.10 ^a^	6.95 ± 0.12 ^a^
TTA (mL/g)	0.64 ± 0.01 ^b^	0.86 ± 0.07 ^a^	0.39 ± 0.02 ^d^	0.49 ± 0.01 ^c^	0.09 ± 0.01 ^e^	0.08 ± 0.02 ^e^
Lactic acid (g/kg)	3.36 ± 0.03 ^c^	3.63 ± 0.03 ^b^	3.68 ± 0.03 ^b^	4.58 ± 0.05 ^a^	n.d. ^2^	n.d. ^2^
Acetic acid (g/kg)	1.70 ± 0.06 ^b^	1.90 ± 0.02 ^a^	n.d. ^2^	n.d. ^2^	n.d. ^2^	n.d. ^2^
**Color parameters**						
L*	77.84 ± 0.06 ^b^	78.00 ± 0.15 ^b^	77.73 ± 0.07 ^c^	78.28 ± 0.03 ^a^	74.45 ± 0.20 ^d^	74.34 ± 0.04 ^d^
a*	5.54 ± 0.06 ^a^	5.55 ± 0.07 ^a^	5.84 ± 0.11 ^b^	5.21 ± 0.06 ^ac^	5.30 ± 0.34 ^a^	6.37 ± 0.02 ^d^
b*	4.89 ± 0.08 ^a^	5.16 ± 0.25 ^abd^	5.42 ± 0.08 ^b^	6.47 ± 0.03 ^c^	5.18 ± 0.26 ^abd^	5.30 ± 0.01 ^d^
WI	76.64 ± 0.05 ^a^	76.73 ± 0.09 ^ac^	76.35 ± 0.09 ^b^	76.74 ± 0.05 ^c^	73.40 ± 0.24 ^d^	73.04 ± 0.03 ^e^

Mean values ± standard deviations. Values in the same row followed by the same superscript letters are not significantly different (*p* > 0.05). n.d. ^1^: no bacterial growth on MRS and GM17 agar; n.d. ^2^: not detected or below 0.3 g/kg.

**Table 4:** Gel point in minutes of oscillatory analysis during fermentation; losses in storage modulus (ΔG′) and loss modulus (ΔG″) in percent between the end of the first oscillatory interval and the end of the second oscillatory interval of the three-interval-thixotropy test; Variables 4–6 of tribological analysis.

	LcTR116	LpMP070	Lbp6.1	Yoflex
Gel point (min)	212.8 ± 1.0 ^c^	306.1 ± 5.4 ^b^	328.3 ± 16.5 ^a^	100.7 ± 8.3 ^d^
ΔG′ (%)	29.80 ± 2.44 ^c^	26.37 ± 2.69 ^d^	36.55 ± 2.41 ^b^	43.52 ± 1.52 ^a^
Δ″ (%)	38.21 ± 2.71 ^c^	40.72 ± 2.45 ^c^	45.09 ± 2.12 ^b^	53.15 ± 1.80 ^a^
Var 4 (m/s) *	0.018 ± 0.003 ^a^	0.023 ± 0.007 ^ab^	0.009 ± 0.000 ^b^	0.020 ± 0.004 ^ab^
Var 5 (-) *	0.018 ± 0.001 ^a^	0.018 ± 0.001 ^a^	0.013 ± 0.001 ^b^	0.020 ± 0.001 ^a^
Var 6 (m/s) *	0.751 ± 0.205 ^ab^	0.633 ± 0.000 ^a^	0.406 ± 0.036 ^b^	0.633 ± 0.000 ^a^

Mean values ± standard deviations. Values in the same row followed by the same superscript letters are not significantly different (*p* > 0.05). * For definition of variables, see Figure 1.

**Table 5:** Content of FODMAPs and sucrose as measured by HPAEC-PAD, in g/100 g.

	Mono-/Disaccharides ^A^			Polyols	Oligosaccharides ^B^		Sucrose
	Glucose	Fructose	EF ^C^	Mannitol	Raffinose/ Stachyose	Verbascose	
UfF ^D^	0.05 ± 0.00 ^a^	4.55 ± 0.05 ^a^	4.50 ^a^	n.d.	0.10 ± 0.00 ^a^	0.05 ± 0.00 ^ab^	0.07 ± 0.00 ^c^
UfS ^E^	0.04 ± 0.00 ^b^	n.d.	-	n.d.	0.010 ± 0.00 ^a^	0.05 ± 0.00 ^ab^	4.60 ± 0.05 ^a^
LcTR116	n.d.	3.42 ± 0.05 ^c^	3.41 ^c^	0.66 ± 0.02 ^b^	0.05 ± 0.00 ^b^	0.05 ± 0.00 ^ab^	n.d.
LpMP070	n.d.	3.51 ± 0.02 ^c^	3.49 ^c^	0.81 ± 0.01 ^a^	0.10 ± 0.00 ^a^	0.04 ± 0.00 ^ab^	n.d.
Lbp6.1	n.d.	4.29 ± 0.02 ^b^	4.27 ^b^	n.d.	0.10 ± 0.00 ^a^	0.05 ± 0.00 ^a^	0.07 ± 0.00 ^c^
Yoflex	0.03 ± 0.00 ^b^	0.06 ± 0.00 ^c^	0.03 ^d^	n.d.	0.10 ± 0.00 ^a^	0.04 ± 0.00 ^b^	4.25 ± 0.02 ^b^

Mean values ± standard deviations. Values in the same row followed by the same superscript letters are not significantly different (*p* > 0.05). n.d. not detected or below 0.02 g/100 g. ^A^ no lactose detected, ^B^ no fructans detected, ^C^ excessive fructose, ^D^ Unfermented (fructose), ^E^ Unfermented (sucrose).

**Table 6:** Contents of antifungal phenolic compounds as measured by UHPLC-UV in mg/kg.

	Unfermented (Fructose)	Unfermented (Sucrose)	LcTR116	LpMP070	Lbp6.1	Yoflex
Phenyllactic acid	n.d.	n.d.	5.52 ± 0.21 ^b^	8.28 ± 1.05 ^a^	4.40 ± 2.14 ^b^	1.55 ± 0.46 ^c^
Coumaric acid	0.27 ± 0.04 ^c^	0.34 ± 0.01 ^c^	0.55 ± 0.01 ^b^	0.66 ± 0.09 ^a^	0.51 ± 0.05 ^b^	0.49 ± 0.01 ^b^
Salicylic acid	0.20 ± 0.03 ^c^	0.24 ± 0.01 ^c^	0.62 ± 0.06 ^a^	0.57 ± 0.07 ^a^	0.41 ± 0.06 ^b^	0.36 ± 0.04 ^b^
Benzoic acid	0.14 ± 0.04 ^b^	0.18 ± 0.01 ^b^	0.27 ± 0.02 ^a^	0.25 ± 0.03 ^a^	0.17 ± 0.05 ^b^	0.09 ± 0.01 ^c^
4-Hydroxybenzoic acid	0.25 ± 0.04 ^e^	0.28 ± 0.00 ^de^	0.39 ± 0.01 ^ab^	0.42 ± 0.05 ^a^	0.35 ± 0.03 ^bc^	0.33 ± 0.01 ^cd^
Vanillic acid	0.10 ± 0.02 ^b^	0.10 ± 0.00 ^ab^	0.01 ± 0.00 ^c^	0.02 ± 0.00 ^c^	0.02 ± 0.00 ^c^	0.13 ± 0.03 ^a^

Mean values ± standard deviations. Values in the same row followed by the same superscript letters are not significantly different (*p* > 0.05). n.d.: not detected.

**Table 7:** Results of the sensory analysis of the fermented samples.

	LcTR116	LpMP070	Lbp6.1	Yoflex
Odor: Overall intensity	4.3 ± 1.8 ^ab^	3.2 ± 2.1 ^b^	5.5 ± 1.5 ^a^	5.9 ± 2.1 ^a^
Odor: Yogurt-like	2.4 ± 1.5 ^b^	2.1 ± 1.0 ^b^	5.2 ± 2.2 ^a^	5.9 ± 2.6 ^a^
Flavor: Overall intensity	6.2 ± 1.2 ^ab^	6.3 ± 1.4 ^a^	5.2 ± 1.7 ^ab^	4.8 ± 1.3 ^b^
Sourness	5.4 ± 1.2 ^b^	7.1 ± 1.3 ^a^	2.6 ± 1.5 ^c^	4.1 ± 1.6 ^bc^
Bitterness	4.2 ± 2.1 ^a^	3.8 ± 2.2 ^a^	2.4 ± 2.0 ^a^	2.9 ± 1.8 ^a^
Beany flavor	4.4 ± 2.3 ^a^	4.5 ± 1.7 ^a^	6.1 ± 2.1 ^a^	4.7 ± 2.2 ^a^
Yogurt-like flavor	2.9 ± 1.3 ^b^	2.9 ± 1.4 ^b^	5.3 ± 2.0 ^a^	5.7 ± 2.0 ^a^
Sour cream-like flavor	4.5 ± 2.3 ^ab^	4.8 ± 2.2 ^a^	2.5 ± 2.0 ^b^	3.7 ± 2.4 ^ab^
Mouthfeel: Firmness	3.9 ± 2.0 ^a^	3.9 ± 1.8 ^a^	3.3 ± 1.8 ^a^	3.8 ± 1.9 ^a^
Mouthfeel: Smoothness	5.7 ± 1.7 ^a^	5.8 ± 1.6 ^a^	6.0 ± 2.2 ^a^	6.4 ± 1.9 ^a^
Aftertaste	5.9 ± 1.9 ^a^	6.2 ± 1.7 ^a^	4.5 ± 2.0 ^ab^	3.6 ± 1.6 ^b^
Overall acceptability	3.7 ± 2.1 ^b^	3.4 ± 1.7 ^b^	5.0 ± 1.9 ^ab^	6.7 ± 1.3 ^a^

Mean values ± standard deviations. Values in the same row followed by the same superscript letters are not significantly different (*p* > 0.05).

## Data Availability

Data are contained within the article.

## Supplementary Materials

The following supporting information can be downloaded at: https://www.mdpi.com/article/10.3390/foods11142013/s1, Figure S1: Questionnaire used for sensory analysis; Figure S2: Stribeck curves of the samples for a sliding speed from 1 × 10^−8^ to 1 m/s.; Table S1: Variables calculated from tribological measurements. For definition of variables, see main manuscript, Figure 1.
