# Supplementary material for: Lentil-Based Yogurt Alternatives Fermented with Multifunctional Strains of Lactic Acid Bacteria—Techno-Functional, Microbiological, and Sensory Characteristics
Source: Foods. 2022 Jul 7;11(14):2013. doi: 10.3390/foods11142013 (PMC9317967; doi:10.3390/foods11142013)
Supplement: Supplementary file 1 [file foods-11-02013-s001.zip › foods-1787981-supplementary.pdf]

foods

Supplementary material

Lentil-based yogurt alternatives fermented with multifunctional strains of lactic acid bacteria – techno-functional, microbiological, and sensory characteristics

Theresa Boeck [1], Lilit Ispiryan [1], Andrea Hoehnel [1], Aylin W. Sahin [1], Aidan Coffey [2,3], Emanuele Zannini [1,*], and Elke K. Arendt [1,3]

1 School of Food and Nutritional Sciences, University College Cork, Cork, Ireland; theresa.boeck@umail.ucc.ie (T.B.); lispiryan@ucc.ie (L.I.); andrea.hohnel@ucc.ie (A.H.); aylin.sahin@ucc.ie (A.W.S.);
2 Department of Biological Sciences, Munster Technological University, Cork, Ireland; aidan.coffey@mtu.ie (A.C.)
3 APC Microbiome Ireland, University College Cork, Ireland; e.arendt@ucc.ie (E.K.A.)
* Correspondence: e.zannini@ucc.ie (E.Z.)

Figure S1: Questionnaire used for sensory analysis.

University College Cork, Ireland
Coláiste na hOllscoile Corcaigh

smart protein

Descriptive Sensory Evaluation

Personal Data

Name: …………………………………… Date: ……………………………………

Information

- Please do not smoke and/or drink coffee 1 hour before the test.
- The samples are lentil protein emulsions fermented with lactic acid bacteria. The following traces of allergens may be present: gluten, peanuts, tree nuts, celery, mustard, eggs, milk, sesame, fish, lupin, soya, sulphites, crustaceans, molluscs.
- All information provided is confidential and the results will not be associated with your name.
- The products have been produced under safe and food grade conditions.

Please confirm that (tick the circle)

- ○ You are aware that the participation in this sensory evaluation is voluntary.
- ○ You are aware that products may contain the allergens reported above.

Guidelines

Please taste the samples for the attributes reported in Table 1. In between each sample, please cleanse your palate with water. Following the example below, please place a line on where you estimate the intensity of the attribute. Adding comments is optional.

Example:

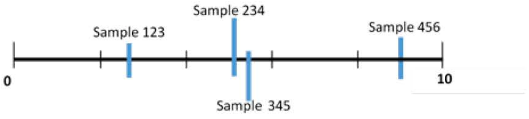

Attribute	Description	Intensity
**Odour – Overall intensity**	The intensity of the odour of the sample.	0 = low, 10 = high
**Odour – Yoghurt-like**	The odour of plain dairy yoghurt.	0 = none, 10 = extreme
**Flavour – Overall intensity**	The intensity of the flavour/taste of the sample	0 = low, 10 = high
**Flavour - Sour**	The basic taste, perceived on the tongue, stimulated by acids, such as citric acid, lactic acid or acetic acid.	0 = none, 10 = extreme
**Flavour – Bitter**	The basic taste, perceived on the tongue, stimulated by substances like caffeine or quinine	0 = none, 10 = extreme
**Flavour - Beany**	The flavour of cooked or canned pulses such as beans or lentils.	0 = none, 10 = extreme
**Flavour - Yoghurt-like**	The flavour of plain dairy yoghurt.	0 = none, 10 = extreme
**Flavour - Sour cream-like**	The flavour of plain, dairy-based sour cream.	0 = none, 10 = extreme
**Mouthfeel - Firmness**	The force required to break up the sample with the tongue.	0 = low, 10 = high
**Mouthfeel - Smoothness**	The degree of melting and dissolving in the mouth, corresponding to creaminess, without the sensation of lumps or particles.	0 = low, 10 = high
**Aftertaste**	Persistence of the taste in the mouth after swallowing.	0 = none, 10 = extreme

Table 1: Attributes for sensory evaluation, and their descriptions.

Sensory Evaluation Fermented Lentil Protein

Figure S2: Stribeck curves of the samples for a sliding speed from 1 x 10^{-08} to 1 m/s. Every curve is the average of triplicates. Error bars have been omitted for clarity

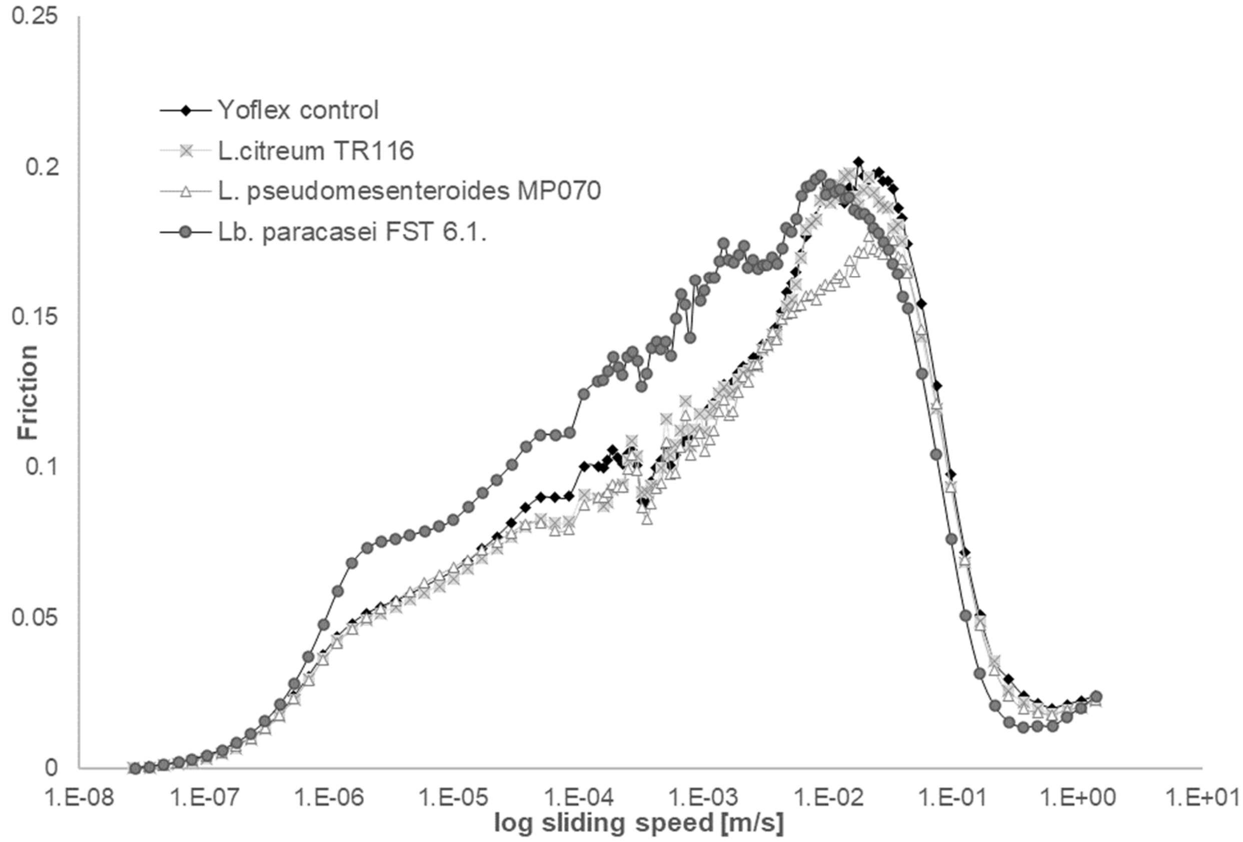

Table S1: Variables calculated from tribological measurements. For definition of variables, see main manuscript, Figure 1.

	**Var 1 [-]**		**Var 2 [-]**		**Var 3 [-]**	
LcTR116	0.114	0.011a	0.092	0.010a	0.203	0.010a
LpMP070	0.11	0.008a	0.086	0.014a	0.178	0.020a
LbpFST6.1	0.053	0.076a	0.112	0.001a	0.197	0.006a
Yoflex control	0.115	0.010a	0.098	0.015a	0.202	0.013a
	**Var 4 [m/s]**		**Var 5 [-]**		**Var 6 [m/s]**	
LcTR116	0.018	0.003a	0.018	0.001a	0.751	0.205ab
LpMP070	0.023	0.007ab	0.018	0.001a	0.633	0.000a
Lbp6.1	0.009	0.000b	0.013	0.001b	0.406	0.036b
Yoflex	0.020	0.004ab	0.02	0.001a	0.633	0.000a

Note: Mean values ± standard deviation. Values in the same column followed by the same superscript letters are not significantly different ($p > 0.05$).
